# Enriched Environment Protects the Optic Nerve from Early Diabetes-Induced Damage in Adult Rats

**DOI:** 10.1371/journal.pone.0136637

**Published:** 2015-08-27

**Authors:** Damián Dorfman, Marcos L. Aranda, Ruth E. Rosenstein

**Affiliations:** Laboratory of Retinal Neurochemistry and Experimental Ophthalmology, Department of Human Biochemistry, School of Medicine/CEFyBO, University of Buenos Aires/CONICET, Buenos Aires, Argentina; Universidade Federal do Rio de Janeiro, BRAZIL

## Abstract

Diabetic retinopathy is a leading cause of reduced visual acuity and acquired blindness. Axoglial alterations of the distal (close to the chiasm) optic nerve (ON) could be the first structural change of the visual pathway in streptozotocin (STZ)-induced diabetes in rats. We analyzed the effect of environmental enrichment on axoglial alterations of the ON provoked by experimental diabetes. For this purpose, three days after vehicle or STZ injection, animals were housed in enriched environment (EE) or remained in a standard environment (SE) for 6 weeks. Anterograde transport, retinal morphology, optic nerve axons (toluidine blue staining and phosphorylated neurofilament heavy immunoreactivity), microglia/macrophages (ionized calcium binding adaptor molecule 1 (Iba-1) immunoreactivity), astrocyte reactivity (glial fibrillary acid protein-immunostaining), myelin (myelin basic protein immunoreactivity), ultrastructure, and brain derived neurotrophic factor (BDNF) levels were assessed in non-diabetic and diabetic animals housed in SE or EE. No differences in retinal morphology or retinal ganglion cell number were observed among groups. EE housing which did not affect the STZ-induced weight loss and hyperglycemia, prevented a decrease in the anterograde transport from the retina to the superior colliculus, ON axon number, and phosphorylated neurofilament heavy immunoreactivity. Moreover, EE housing prevented an increase in Iba-1 immunoreactivity, and astrocyte reactivity, as well as ultrastructural myelin alterations in the ON distal portion at early stages of diabetes. In addition, EE housing avoided a decrease in BDNF levels induced by experimental diabetes. These results suggest that EE induced neuroprotection in the diabetic visual pathway.

## Introduction

Environmental enrichment refers to a housing condition that allows enhanced sensory, cognitive and motor stimulation, relative to standard laboratory conditions [1,2]. In an enriched environment (EE), animals are housed in large cages containing a variety of objects that are daily changed, and running wheels for voluntary physical exercise, as well as increased opportunities for social interaction [2–4]. In the central nervous system (CNS), physiological remodeling or repair processes are strongly influenced by experience-dependent mechanisms [3–5]. EE housing enhances learning and memory, and improves compensatory processes in the damaged CNS [3, 6–8]. The exposure to EE accelerates the development of the visual system, and enhances visual cortex plasticity both during development and adulthood [9]. Adult rats housed in a complex environment have significantly more synapses per neuron in the visual cortex compared to control animals [10]. Moreover, it has been demonstrated that the exposure of adult amblyopic rats to EE promotes a complete recovery of visual acuity and ocular dominance, probably through a brain derived neurotrophic factor (BDNF)-dependent mechanism [11], and that post-ischemic environmental enrichment housing protects the adult rat retina from acute ischemic damage [12]. In addition, prolonged exposure to EE from birth of rd10 mice, a mutant strain undergoing progressive photoreceptor degeneration mimicking human retinitis pigmentosa, induces remarkable therapeutic effects on the visual system [13]. These findings highlight the potential of EE as a promising non-invasive strategy to promote recovery of normal sensory functions in different animal models of neurodegeneration in the adult CNS.

Diabetic retinopathy (DR), one of the most serious complications of diabetes, is a leading cause of reduced visual acuity and acquired blindness. Almost all individuals with type 1 diabetes mellitus, and more than 60% of individuals with type 2 diabetes mellitus have some degree of retinopathy after 20 years of diabetes [14]. In inadequately controlled patients, the retinal microvasculature is constantly exposed to hyperglycemia, which provokes vascular damage and leakage, edema, capillary basement membrane thickening, neovascularization, hemorrhage, ischemia, and neuroglial alterations [15, 16]. Visual function disorders have been demonstrated in diabetic patients with very early stages or even before the onset of retinopathy [17]. In that context, it was shown that diabetes induces nonvascular cell death and retinal neurodegeneration [15]. Moreover, diabetes provokes early changes in the visual signal transmission and its central processing, which take place before the appearance of the first ophthalmoscopically detectable signs of DR [18–20]. Significant loss of contrast sensitivity and color vision impairments have been described in patients with type 1 diabetes whom had no evidence of retinopathy [21–24]. Loss or remodeling of neurons in the retina or the visual pathway might account for the early reduction of visual function in diabetes. Streptozotocin (STZ)-induced diabetes is a well validated model of type 1 diabetes in rodents (reviewed in [25]). We have demonstrated a significant axon loss, a large increase in astrocyte reactivity, and myelin alterations only in the distal (but not the proximal, near to the retina) portion of the optic nerve (ON) at 6 weeks of experimental diabetes induced by STZ [26]. Since at this time point (i.e., at 6 weeks after STZ injection), no substantial structural alterations are evident in the retina, or the main retinal synaptic target in rodents, the superior colliculus (SC), these results suggest that axoglial alterations at the distal (close to the chiasm) portion of the ON could be the first structural change in the diabetic visual pathway [26]. Recently, we have demonstrated that EE housing prevents the electroretinographic dysfunction, and preserves inner retina synapses, and blood retinal barrier integrity at early stages of experimental diabetes induced by STZ in adult rats [27]. Having demonstrated significant neurodegenerative changes in the distal portion of the ON, and a protective effect of EE on retinal injury at early stages of experimental diabetes, the aim of the present report was to explore whether EE is able to protect the ON from axoglial alterations induced by experimental diabetes.

## Materials and Methods

### Ethics Statement

All animal procedures were in strict accordance with the ARVO Statement for the Use of Animals in Ophthalmic and Vision Research. The ethic committee of the School of Medicine, University of Buenos Aires (Institutional Committee for the Care and Use of Laboratory Animals, (CICUAL)) approved this study, and all efforts were made to minimize animal suffering.

### Animals

Adult male *Wistar* rats (average weight 300 ± 50 g) were housed in a standard animal room with food and water *ad libitum*, under controlled conditions of humidity and temperature (21 ± 2°C). The room was lighted by fluorescent lights (200 lux), that were turned on and off automatically every 12 h (on from 8.00 AM to 8.00 PM). For the control group (standard environment, (SE)), animals (2 per cage) were housed in standard laboratory cages (33.5 x 45 x 21.5 cm). For enriched environment (EE) housing, six animals at a time were housed in big metallic cages (46.5 x 78 x 95 cm), containing four floors and several food hoppers, water bottles, running wheels, tubes, ramps and differently shaped objects (balls, ropes, stones) daily repositioned, and fully substituted once a week, as previously described [27]. Particular care was taken not to repeat cage arrangement and object availability during the experiments. Food and water were offered *ad libitum*, but the location of the hoppers and bottles was daily changed in order to stimulate exploratory conduct. Cages were cleaned twice a week at the same time and by the same protocol to that used for standard cage cleaning.

For diabetes induction, a single intraperitoneal injection of STZ (60 mg/kg in 0.1 M citrate buffer, pH 4.5) was performed, whereas control rats received an equal volume of citrate buffer. Animals were examined 3 days after injections with a glucose meter (Bayer, Buenos Aires, Argentina), and those with glycemia greater than 350 mg/dl were considered diabetic, and caged in SE or EE. The body weight and plasma glucose levels were weekly monitored. A total number of 86 animals were used for the experiments, distributed as follows: 24 animals for anterograde transport study, 48 animals for histological and immunohistochemical studies, and 24 animals for semithin and ultrathin sections analysis.

### Cholera toxin β-subunit injection

Rats were anesthetized with ketamine hydrochloride (150 mg/kg) and xylazine hydrochloride (2 mg/kg) administered intraperitoneally, and a drop of 0.5% proparacaine was topically administered for local anesthesia. Four microliters of 0.1% cholera toxin β-subunit (CTB) conjugated to Alexa 488 dye (Molecular Probes Inc., Eugene, OR, USA) in 0.1 mol/L PBS (pH 7.4) were injected into the vitreous, using a 30-gauge Hamilton syringe (Hamilton, Reno, NV, USA), as previously described [26]. The injections were applied at 1 mm from the limbus, and the needle was left in the eye for 1 minute to prevent volume loss. Three days after injection, rats were anesthetized as previously described and intracardially perfused with saline solution, followed by a fixative solution, containing 4% formaldehyde in 0.1 mol/L PBS (pH 7.4). Brains were carefully removed, post-fixed overnight at 4°C, and immersed in a graded series of sucrose solutions for cryoprotection. Coronal sections (40 μm) were obtained using a freezing microtome (Leica Microsystems, Buenos Aires, Argentina). Sections were mounted on charged slides (Erie Scientific Company, New Hampshire, USA), with antifade medium (Vectashield, Vector Laboratories, Burlingame, CA, USA), and viewed with a fluorescence microscope (BX-50, Olympus, Tokyo, Japan) connected to a digital camera (3CCD, Sony, Tokyo, Japan). Images were obtained using ImagePro Plus software (Optimus, Media Cybernetics, Silver Spring, MD, USA). Every other coronal section from the beginning to the end of the SC (approximately 30 sections) was used for the SC retinorecipient area reconstruction, using Matlab (The MathWorks Inc., Natick, MA, USA). Digital images were converted to 8-bit grayscale, and the optic density of CTB staining was calculated. The total length was measured and divided into bins (4 μm), from the medial to lateral region. CTB quantification was performed by dividing the total pixel area by CTB+ pixels. Finally, a colorimetric thermal representation was applied (from 0% = blue to 100% = red). The number of sections and the thickness (2X) were used for a final reconstruction of the retinal projection to the SC.

### Tissue processing

Anesthetized rats were perfused as already described. Then, the eyeballs with the intraorbital (near the retina, proximal) ON portion and the brain with the intracranial (near the chiasm, distal) ON portion were carefully removed and immersed for 24 h in the same fixative. After dehydration, eyecups and ONs were included in paraffin wax, and transversal sections (5 μm) were obtained with a microtome (2125 RTS, Leica Biosystems, Buenos Aires, Argentina), and mounted on charged slides (Erie Scientific Company, New Hampshire, USA). For histological analysis, retinal sections were stained with hematoxylin and eosin (H&E). Microscopic images were digitally captured with a microscope (Eclipse E400, Nikon, Tokyo, Japan); 6-V halogen lamp, 20 W, equipped with a stabilized light source) and a camera (Coolpix s10; Nikon; Abingdon, VA, USA) and the total retinal thickness (in μm) and the number of cells in the ganglion cell layer (GCL) were assessed. Measurements (400X) were obtained at 1 mm dorsal and ventral from the optic disc. The number of cells in the GCL was counted along the whole retina section (400X). For each eye, results obtained from four separate sections were averaged, and the mean of 5 eyes was recorded as the representative value for each group.

### Immunofluorescence

Antigen retrieval was performed in paraffin sections, by heating (90°C) slices for 30 minutes in citrate buffer (pH 6.3). Sections were preincubated with 2% normal horse serum for 1 h, and then were incubated overnight at 4°C with primary antibodies. A mouse monoclonal anti-phosphorylated neurofilament heavy (NFHp) (1:1000; catalogue #: ab24570, Abcam, MA, USA), a goat polyclonal anti-ionized calcium binding adaptor molecule 1 (Iba-1) (1:1000; catalogue #: ab5076, Abcam, MA, USA), a mouse monoclonal anti-glial fibrillary acidic protein (GFAP) conjugated to Cy3 (1:1200; catalogue #: c9205, Sigma Chemical Co., St. Louis, MO, USA), a mouse monoclonal anti-brain derived neurotrophic factor (BDNF) (1:50; catalogue #: sc-546, Santa Cruz Biotechnology, Buenos Aires, Argentina), and a rabbit polyclonal anti-myelin basic protein (MBP, 1:1000; generously donated by Dr. Campagnoni, Mental Retardation Research Center, University of California, Los Angeles, CA, USA) were used. After several washings, secondary antibodies were added, and sections were incubated for 2 h at room temperature. Regularly, some sections were treated without the primary antibodies to confirm specificity. Nuclei were stained with DAPI, mounted using antifade medium (Vectashield, Vector Laboratories, CA, USA), and viewed using a fluorescence microscope (BX-50, Olympus, Tokyo, Japan), connected to a video camera (3CCD; Sony, Tokyo, Japan) attached to a computer running image analysis software (Optimus, Media Cybernetics, Silver Spring, MD, USA). Comparative digital images from different samples were obtained using identical exposure time, brightness, and contrast settings.

### Quantification of NFHp(+)-, GFAP(+), and Iba1-(+) area

The NFHp(+), GFAP(+), and Iba1(+) area was measured in ON sections and expressed as percentage of an identical total area. In addition, NFHp(+) spots were measured. NIH ImageJ Software (National Institutes of Health, Bethesda, Maryland; http://imagej.nih.gov/ij/) was used to quantify the intensity for each parameter. The mean of 5 ONs was recorded as the representative value for each group.

### Quantification of BDNF-immunoreactivity

The BDNF(+) area was measured in ON sections, and expressed as percentage of the total area. NIH ImageJ Software (National Institutes of Health, Bethesda, Maryland; http://imagej.nih.gov/ij/) was used to quantify BDNF intensity in identical rectangular areas of ON longitudinal sections (400X). Results obtained from four sections were averaged, and the mean of 5 eyes was recorded as the representative value.

### Retinal ganglion cell quantification

Flatmount retinas were obtained as previously described [28], and incubated overnight with a polyclonal goat anti-Brn3a antibody (1:500; catalogue #: sc31984, Santa Cruz Biotechnology, Buenos Aires, Argentina), after several washings, secondary antibodies were added, and sections were incubated for 2 h at room temperature. Images (200X; area corresponding to 0.1 mm^2^) from 4 different quadrants from the central and peripheral retina were captured, and the mean of 20 images was considered as the representative value, and expressed as the total number of Brn3a(+) cells in 2 mm^2^.

### Optic nerve analysis

Morphometric analysis of the ON was performed as previously described [26]. Briefly, under deep anesthesia, animals were intracardially perfused with saline, containing 0.5 ml heparin and 2.4% sodium nitroprusside as vasodilator, followed by a fixative solution containing 2% glutaraldehyde and 4% formaldehyde in 0.1 M PBS (pH 7.4). Eyes were carefully removed and the proximal (2 mm after the ON head) and distal (2 mm before the optic chiasm) portions were obtained, as previously described. After several washings, the samples were post-fixed in 1% osmium tetroxide in sodium phosphate buffer for 1 h. Dehydration was accomplished by gradual ethanol series, and tissue samples were embedded in epoxy resin for semithin or ultrathin sectioning. Semithin sections (0.5 μm) were obtained using an ultramicrotome (Ultracut E, Reichert-Jung, Salzburg, Austria), stained with toluidine blue-borax, and used for morphometric analysis. Light microscopic images were digitally captured using a Nikon Eclipse E400 microscope via a Nikon Coolpix s10 camera (Nikon, Tokyo, Japan). ON total area was calculated from light microphotographs of transverse semithin sections obtained with a final magnification of 20X. Images at high magnification (1000X) of the ON were obtained (area corresponding to 0.01 mm^2^) and the number of the axons were quantified taking the internal margin of myelin sheath as a reference, using ImageJ software version 1.42q (NIH, Bethesda, MD, USA). For each sample, 5 different images were quantified, and the mean of 6 ONs from different animals were averaged and taken as the representative value for each group. All the images obtained were assembled and processed using Adobe Photoshop SC (Adobe Systems, San Jose, CA, USA) to adjust the brightness and contrast. No other adjustments were made.

### Electron microscopy

Ultrathin sections (70 nm) were obtained, mounted on 200 *mesh* grids, and stained with uranyl acetate 2% in ethanol 70%) and Reynold´s lead citrate. Finally, sections were viewed and photographed (3000X, 12000X, and 50000X) using a transmission electron microscope (Zeiss EM 10 C; Zeiss, Oberkochen, Germany), equipped with a digital camera (ES1000W, Gatan, Pleasanton, CA, USA) for morphologic analysis.

### Statistical analysis

The results were analyzed by two-way ANOVA in a completely randomized design (diabetes and EE). Comparisons were made with the Tukey’s test. Results were considered significant at *P* < 0.05.

## Results

**Fig 1:**
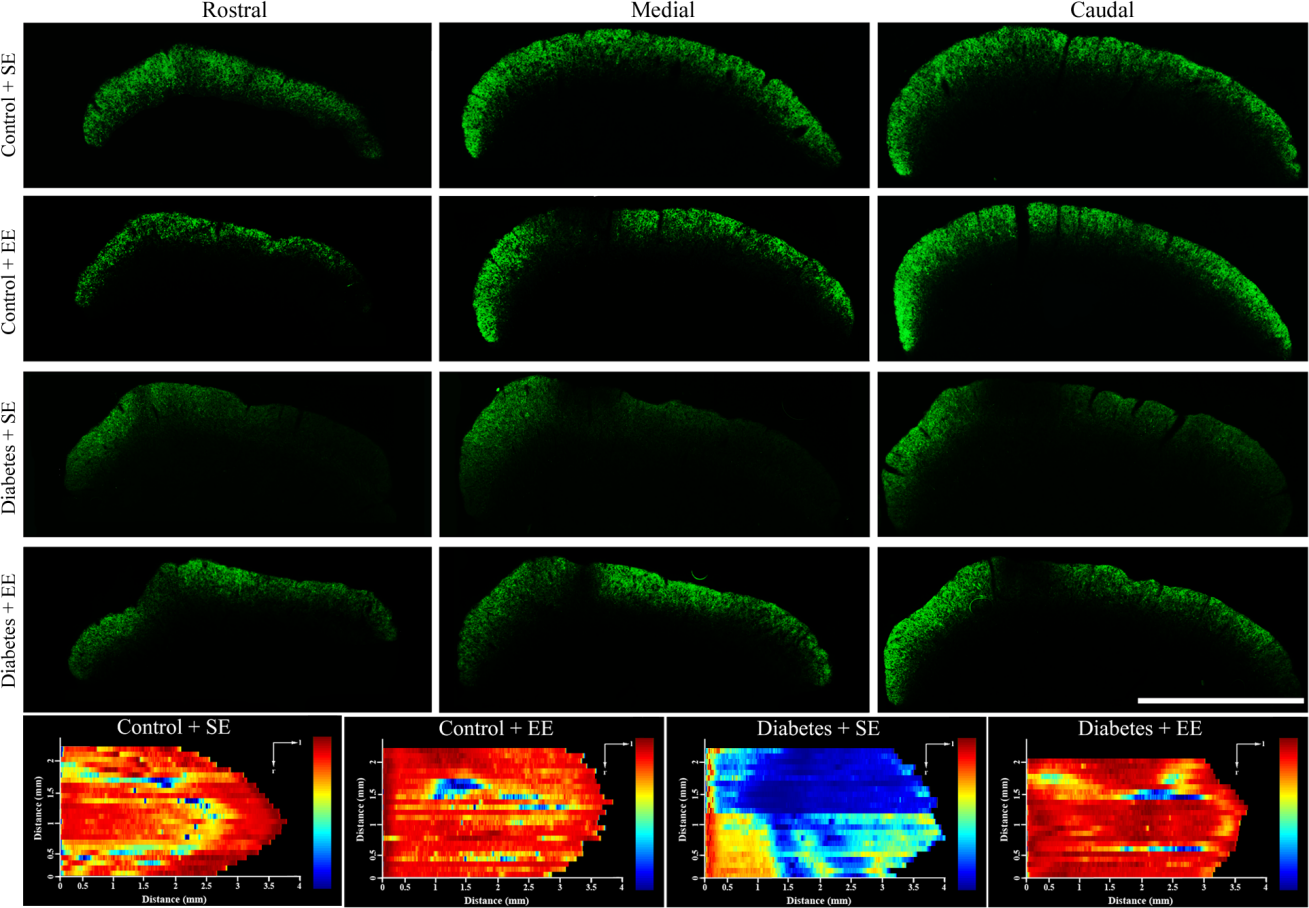
Effect of EE housing on CTB anterograde transport. Photomicrographs showing retinal projections in the superficial layers of the SC from control or diabetic rats housed in SE or EE. Three representative sections (rostral, medial, and caudal) for each group are shown. In SE-housed animals that were diabetic for 6 weeks, a clear reduction in the density of retinal terminals and zones of no CTB staining were found, particularly in the lateral area. EE housing which was ineffective in non-diabetic animals, preserved CTB anterograde transport in diabetic animals. Dorsal views of a retinotopic SC map reconstruction, representative of 6 animals per group are also shown. Scale bar = 1 mm.

The average body weight and glycemia of SE- or EE-housed animals at 6 weeks after the injection of vehicle or STZ are shown in Table 1. A significant weight loss and an increase in blood glucose levels were observed in STZ-treated rats, as compared with vehicle-injected rats. EE housing did not change these parameters in control or diabetic animals. The active anterograde transport from retinal ganglion cells (RGCs) to the SC at 6 weeks of diabetes induction was analyzed using CTB. In rodents, virtually all RGCs project to the *stratum zonale* and the *stratum griseum superficiale* of the contralateral SC. In non-diabetic animals, intensely CTB-stained retinal terminals were observed in the SC (Fig 1). After 6 weeks of diabetes onset, a clear reduction in CTB-staining was observed in the SC from animals housed in SE, whereas in EE-housed diabetic animals, the deficit in CTB transport induced by experimental diabetes was prevented (Fig 1). EE housing did not affect CTB anterograde transport in non-diabetic animals.

**Fig 2:**
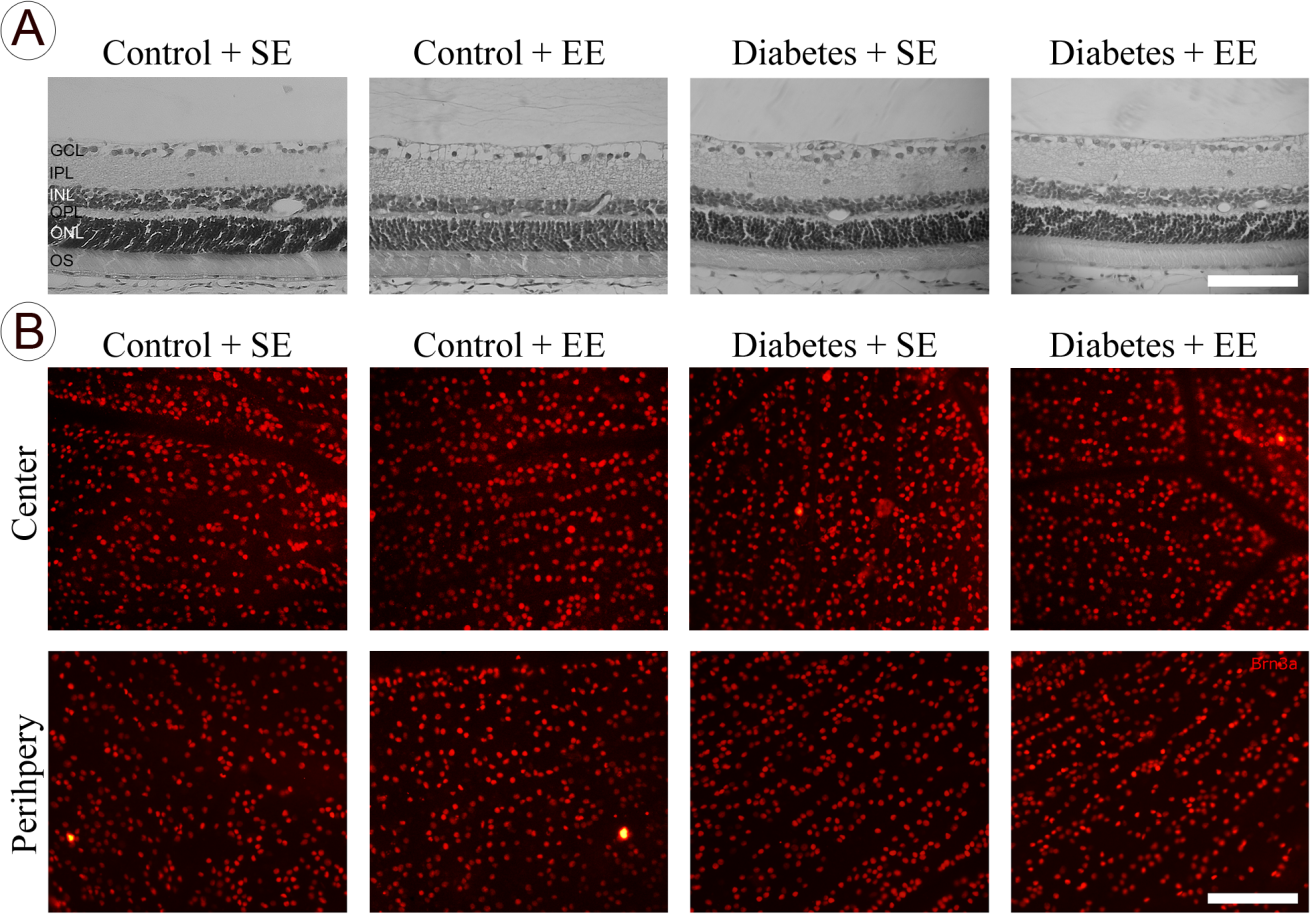
Retinal histology and RGC analysis. Panel A: Representative photomicrographs showing the histologic appearance of retinas from control or diabetic rats housed in SE or EE for 6 weeks (hematoxylin and eosin staining). No obvious differences in the retinal structure were observed among groups. OS, photoreceptor outer segments; INL, inner nuclear layer; IPL, inner plexiform layer; ONL, outer nuclear layer; OPL, outer plexiform layer; GCL, ganglion cell layer. Panel B: Shown are representative photomicrographs of Brn3a immunostaining in flat-mounted retinas in center and periphery from 5 animals/group. Scale bar: 50 μm.

At 6 weeks after vehicle or STZ injection, Brn3a-immunoreactivity in the central and peripheral retina did not differ among groups, as shown in Fig 2. In addition, no differences in the total retinal thickness, GCL cell number, and Brn3a(+) cell number were observed among groups (Table 2). EE housing did not affect these parameters in non-diabetic rats.

**Table 1:** Average body weight and blood glucose concentration in SE- or EE-housed animals.

	Average of body weight (g)				Average of blood glucose concentration (mg/dl)			
	Control + SE	Control + EE	Diabetes + SE	Diabetes + EE	Control + SE	Control + EE	Diabetes + SE	Diabetes + EE
**Before vehicle or STZ injection**	397 ± 1	392 ± 4	398 ± 9	393 ± 16	114 ± 3	112 ± 2	112 ± 3	115 ± 2
**6 weeks after vehicle or STZ injection**	486 ± 10	498 ± 8	326 ± 8 **	322 ± 5**	111 ± 2	115 ± 3	572 ± 21**	553 ± 28**

Body weight and blood glucose levels in vehicle- or STZ-injected animals housed in SE or EE. STZ induced a significant decrease in body weight and an increase in blood glucose levels, which did not differ between SE- and EE-housed animals. Data are mean ± SEM (n = 12 animals per group).

**: *P* < 0.01 vs. non-diabetic animals in SE, by Tukey’s test.

**Table 2:** Retinal morphometric analysis at 6 weeks of diabetes.

	Control + SE	Control + EE	Diabetes + SE	Diabetes + EE
**Total retinal thickness**	120.4 ± 2	119.9 ± 1.9	121.9 ± 2.5	120.8 ± 2.6
**Cell number in the GCL**	15.3 ± 0.4	15.4 ± 0.4	15.4 ± 0.8	15.9 ± 0.7
**Brn3a(+) number / 2 mm** ^**2**^	4147 ± 190	4213 ± 189	4014 ± 150	4042 ± 200

Quantification of total retinal thickness, GCL cells, and Brn3a(+) cells. These parameters did not differ among control or diabetic animals housed in SE or EE. Data are mean ± SEM (n = 5 animals per group).

**Fig 3:**
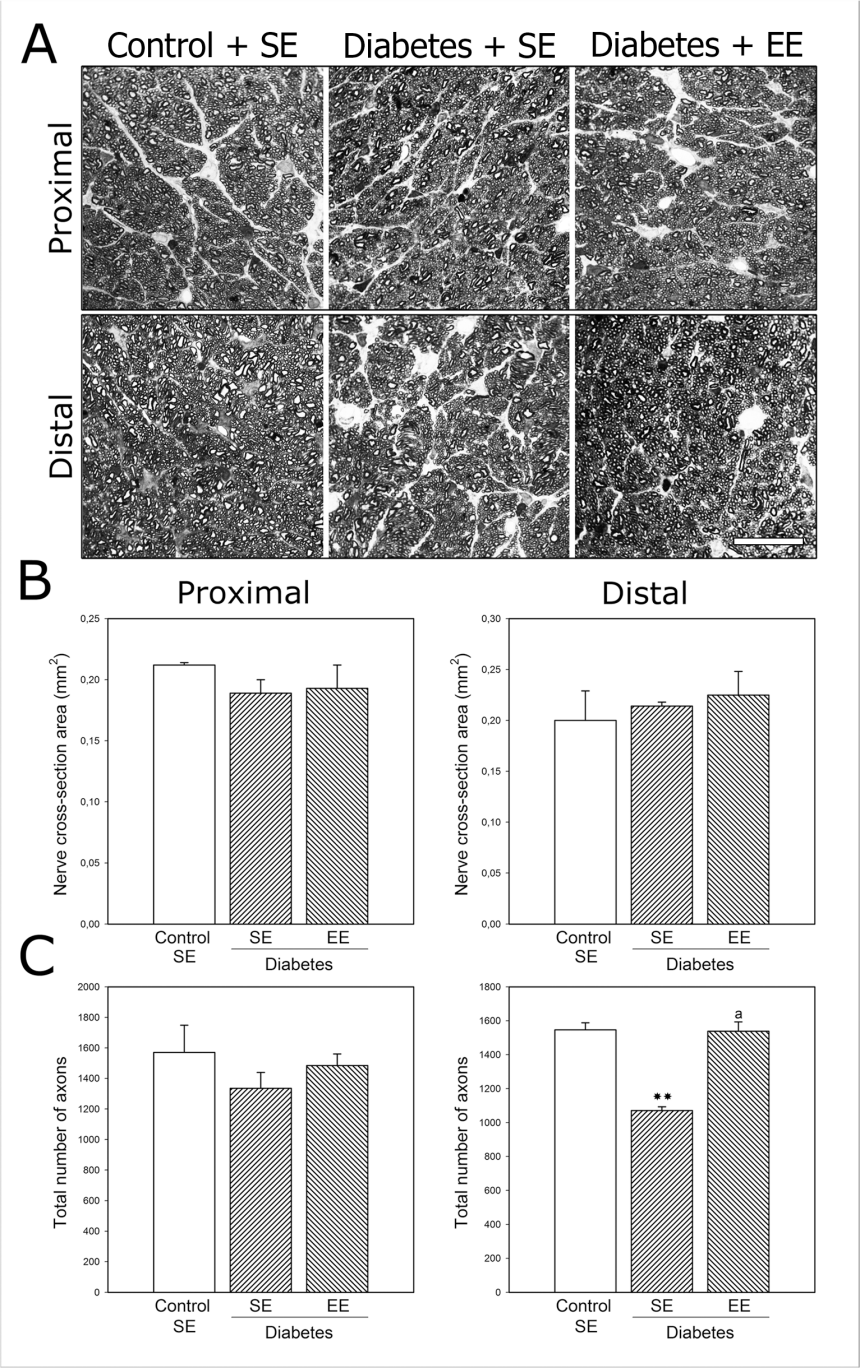
Analysis of ON axons. Panel A: Light micrographs of semithin transverse sections at the proximal and distal myelinated portions of the ON from a non-diabetic rat housed in SE, a diabetic rat housed in SE, and a diabetic rat housed in EE (toluidine blue staining). Scale bar = 25 μm. Panel B: No differences in the ON transversal area were found among groups. Panel C: Quantification of axon number. In SE-housed animals, experimental diabetes induced a significant decrease in the axon number at the distal (but not proximal) portion of the ON which was prevented by EE housing. Data are mean ±SEM (n: 6 animals per group), **P < 0.01 vs. SE-housed non-diabetic animals, a: *P* < 0.01 vs. SE-housed diabetic animals, by Tukey’s test.

**Fig 4:**
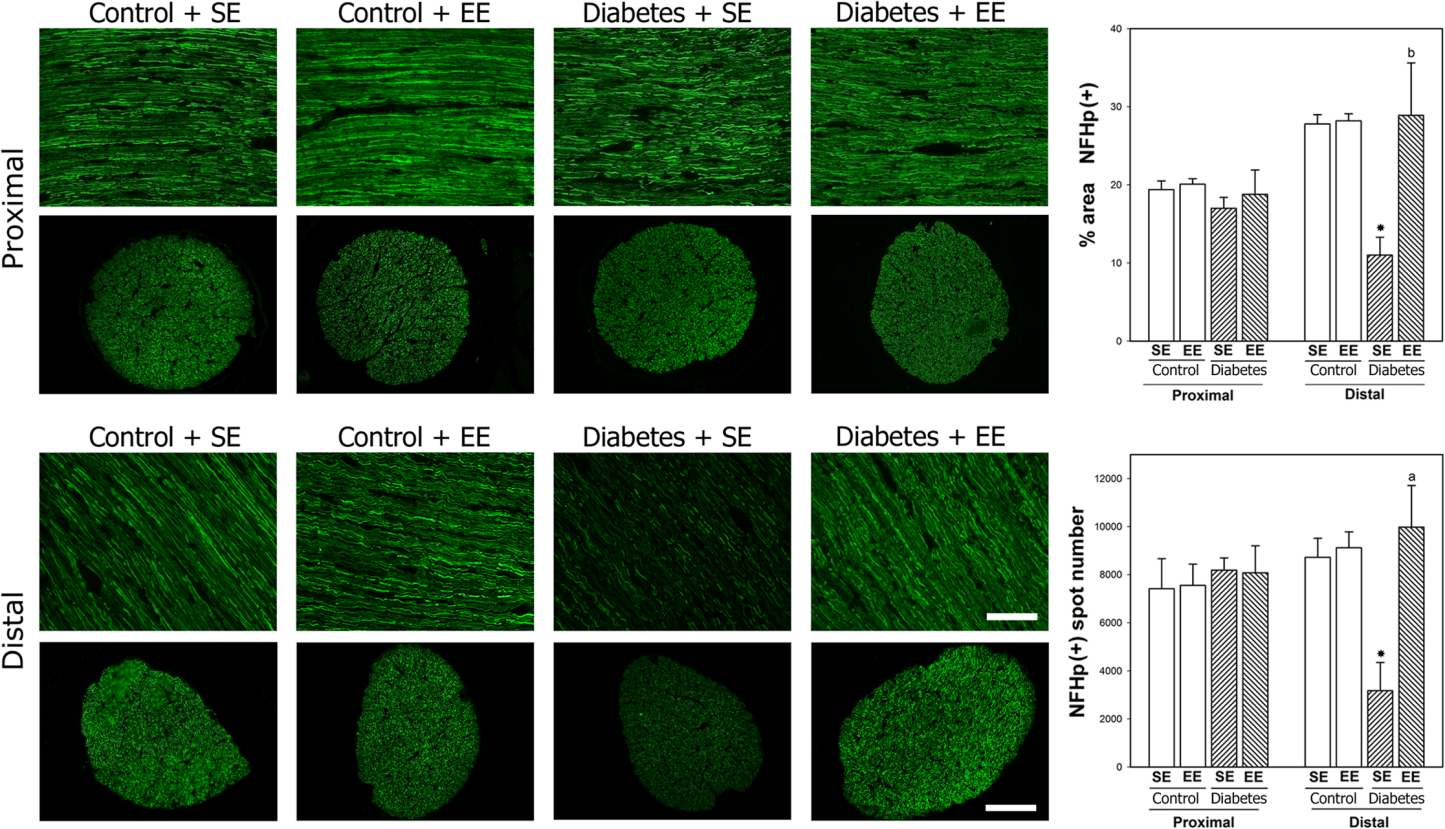
NFHp immunolabeling. Upper panel: Representative photomicrographs showing NFHp immunostaining in longitudinal and transversal sections of the proximal portion of ON from non-diabetic and diabetic animals housed in SE or EE. Lower panel: NFHp immunostaining in the distal portion of the ON. Scale bar: superior panel = 50 μm, inferior panel = 100 μm. Right panel: Quantification of the NFHp(+) area and NFHp(+) spot number in the proximal and distal ON. In SE-housed animals, experimental diabetes induced a significant decrease in these parameters in the distal (but not proximal) ON which was prevented by EE housing. NFHp-immunostaining in the proximal and distal ON did not differ between non-diabetic animals housed in SE or EE. Data are mean ±SEM (n: 5 animals per group), *P < 0.05, **P < 0.01 vs. SE-housed non-diabetic animals, b: *P* < 0.05, a: *P* < 0.01 vs. SE-housed diabetic animals, by Tukey’s test.

ON axons were analyzed by toluidine blue staining, as shown in Fig 3. At 6 weeks of experimental diabetes, a significant reduction in the axon number (but not in nerve cross-section area) was observed at the distal (but not proximal) portion of the ON. In EE-housed diabetic animals, the axon number at the distal portion of the ON was similar to that observed in non-diabetic animals housed in SE. No differences in the proximal portion of the ON were observed among groups, and EE housing of non-diabetic rats did not affect these parameters (data not shown). To further investigate the effect of EE on ON axons, NFHp-immunoreactivity was analyzed in the proximal and distal ON from diabetic animals housed in SE or EE. As shown in Fig 4, intense NFHp-immunoreactivity was observed in longitudinal and transversal sections of the proximal and distal portions of ONs from non-diabetic animals housed in SE or EE. A significant decrease in NFHp-immunoreactivity in distal (but not proximal) ON portion was observed in SE-housed diabetic animals, whereas EE housing prevented the effect of experimental diabetes on this parameter.

**Fig 5:**
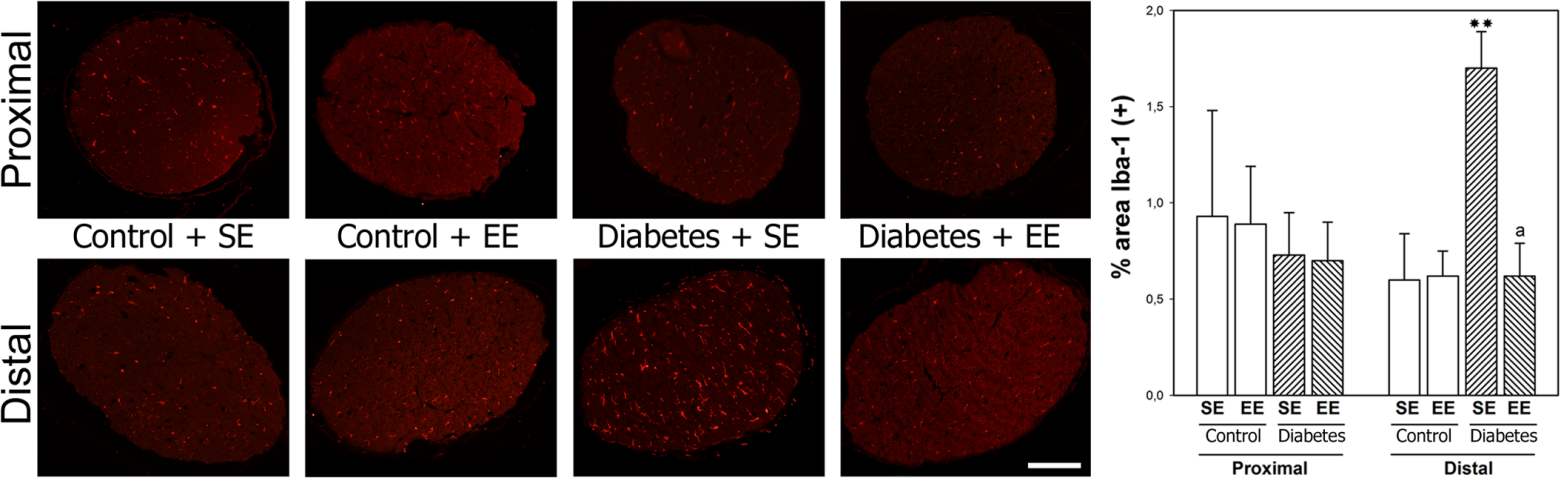
Microglia/macrophages in the ON. Representative photomicrographs showing Iba-1 immunostaining in cross-sections of proximal and distal portions of the ON from SE or EE-housed rats at 6 weeks after vehicle or STZ injection. Analysis of Iba-1(+) area. Experimental diabetes induced a significant increase of Iba-1(+) area in the distal ON from SE (but not EE) housed animals. No changes in Iba-1(+) area were observed in the proximal ON among groups. Data are mean ± SEM (n: 5 animals per group), **P < 0.01 vs. SE-housed non-diabetic animals, a: P < 0.01 vs. SE-housed diabetic animals, by Tukey’s test. Scale bar = 50 μm.

Proximal and distal ON microglia/macrophages were analyzed by Iba-1 immunostaining, as shown in Fig 5. At 6 weeks after STZ injection, an increase in Iba-1 immunoreactivity was observed in transversal sections of the distal (but not proximal) portions of ON from SE-housed diabetic as compared with non-diabetic animals, whereas EE housing decreased the effect of experimental diabetes on this parameter. EE housing of non-diabetic animals did not affect Iba-1 immunoreactivity in the proximal and distal ON.

**Fig 6:**
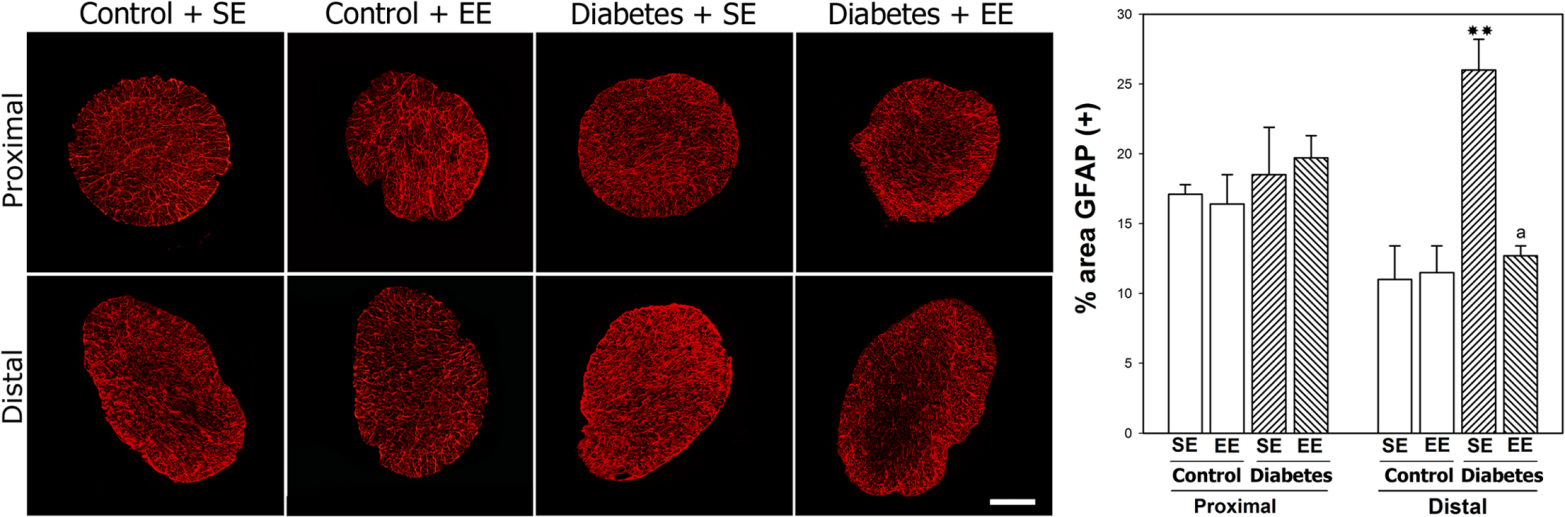
Astrocyte analysis in the ON. Representative GFAP-immunostaining in transverse sections of proximal and distal ON from a non-diabetic rat housed in SE or EE, and a diabetic rat housed in SE or EE. In the distal (but not proximal) ON from SE-housed diabetic rats, the area occupied by astrocytes (GFAP(+) area) was significantly increased. EE housing prevented the effect of experimental diabetes on GFAP immunoreactivity. Data are mean ± SEM (n: 5 animals per group), **P < 0.01 vs. SE-housed non-diabetic animals, a: *P* < 0.01 vs. SE-housed diabetic animals, by Tukey’s test. Scale bar = 100 μm.

**Fig 7:**
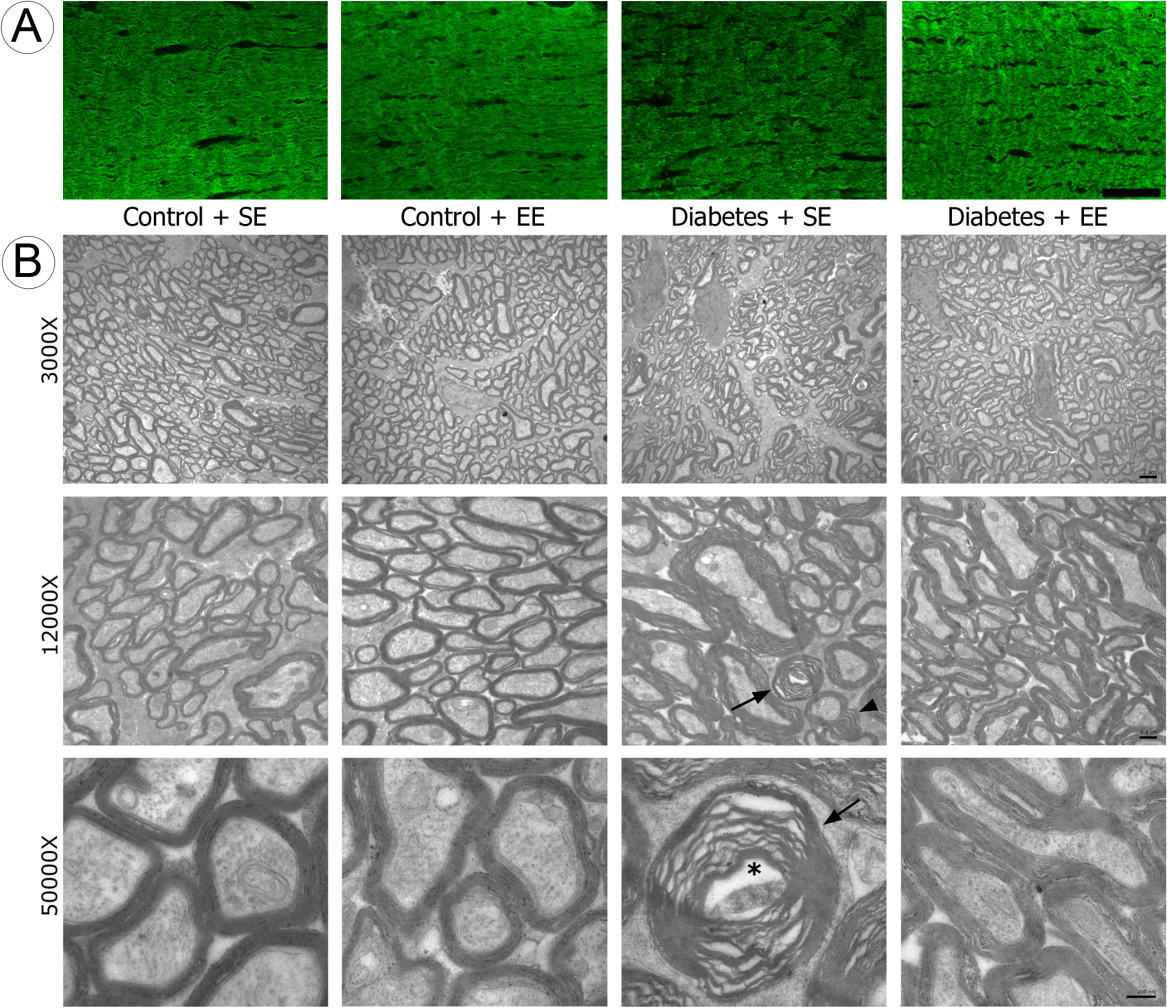
Myelination and ultrastructural analysis of the distal ON. Panel A: MBP-immunoreactivity was examined in the distal portion of the ON from all the experimental groups. A slight disorganization of MBP-immunostaining was observed in SE-housed diabetic animals as compared with SE- or EE-housed non-diabetic animals or diabetic animals housed in EE. Shown are images representative of 5 animals per group. Scale bar = 50 μm. Panel B: Transverse sections of the distal ON from all the experimental groups. In SE- and EE-housed non-diabetic animals, compact bundles of myelinated axons were observed, whereas in the ON from SE-housed diabetic animals, myelin was highly disorganized (arrow head), and frequent lamellar membranous bodies (arrow) were observed. EE housing prevented these alterations in diabetic animals. Shown are images representative of 6 animals per group. Scale bar superior panel = 2 μm; middle panel = 0.5 μm, inferior panel = 200 nm.

To investigate the effect of EE housing on ON astrocytes, GFAP-immunoreactivity was analyzed in the proximal and distal ON portion from control or diabetic rats housed in SE or EE. A significant increase in GFAP-immunoreactivity was observed in the distal ON from SE-housed diabetic rats as compared with SE- or EE-housed non-diabetic animals, whereas EE housing prevented the increase of this parameter in diabetic animals (Fig 6). No changes in GFAP-immunoreactivity were observed in the proximal ON. Fig 7 shows MBP-immunostaining in ON sections from non-diabetic and diabetic animals housed in SE or EE, and ultrastructural analysis of the distal ON from all the experimental groups. A slight disorganization of myelin, with some demyelinated zones were observed in the distal ON from diabetic animals housed in SE, whereas in diabetic animals housed in EE, MBP-immunoreactivity was similar to that found in non-diabetic animals housed in SE or EE. At ultrastructural level, compact bundles of myelinated axons were observed in ONs from non-diabetic animals housed in SE or EE. In the distal ON from SE-housed diabetic animals, myelin was highly disorganized, and frequent lamellar membranous bodies were observed. EE housing prevented these ultrastructural alterations in diabetic animals (Fig 7).

**Fig 8:**
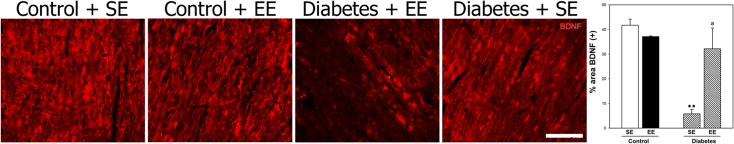
BDNF immunoreactivity in the distal ON after 6 weeks of diabetes. In diabetic animals housed in SE, a significant decrease in BDNF immunostaining was observed in the distal portion of the ON, whereas in the ON from diabetic animals housed in EE, BDNF immunoreactivity was similar to that found in non-diabetic animals kept in SE or EE. Shown are photographs representative of 5 ONs from different animals per group. Scale bar = 50 μm.

BDNF-immunoreactivity in the distal ON portion was evaluated at 6 weeks after STZ injection. In SE-housed animals, diabetes induced a significant decrease in BDNF-immunoreactivity, whereas in EE-housed diabetic animals, BDNF-immunoreactivity was similar to that observed in the ON from non-diabetic animals housed in SE or EE, as shown in Fig 8.

## Discussion

The exposure to EE is known to produce functional recovery after various types of CNS lesions [4, 29, 30]. We have previously shown that EE housing protects the adult retina against acute ischemic [12], and early diabetic [27] damage. However, the beneficial effect of EE housing against ON alterations induced by chronic damage such as STZ-induced experimental diabetes was not previously examined. In a previous report, we have shown that in STZ-induced diabetes, axonal alterations at the distal portion of the ON precede RGC loss [26]. The present results indicate that EE housing, which had no effects *per se* in non-diabetic animals, prevented axoglial alterations of the ON observed at early stages of experimental diabetes. Remarkably, the protective effect of EE was independent from the metabolic profile, as suggested by the fact that EE did not affect the STZ-induced weight loss and hyperglycemia.

DR is a major sight-threatening disease, and accumulating evidence indicates that glial activation and neuron injury occur at early stages of the disease [31,32]. In addition, histopathological findings in 3-month diabetic rats demonstrate vacuolization of myelin, segmentation loss, and proliferation of astrocytes in the ON [33]. As previously described, a loss of connectivity between the retina and its main central target in rodents (the SC) occurred in SE-housed animals at early stages of diabetes [26], whereas EE housing prevented the decrease in the density of CTB-labeled axon terminals in the SC from diabetic animals. In agreement with these results, it has been demonstrated that EE promotes an increase in retinal CTB transport after deafferentation of the SC in adult rats [34]. Although no obvious changes in retinal morphology or in the number of RGCs were observed in the central and peripheral retina from SE-housed animals that had been diabetic for 6 weeks, a significant decrease in axon number occurred in the distal (but not proximal) portion of the ON, whereas the exposure to EE prevented the decrease in axon number. As the main component of the neuron cytoskeleton, neurofilament protein mainly distributes in the cell body and processes of neurons, and plays an important role in maintaining the normal morphology and structure of neurons [35]. Although these proteins are primarily dephosphorylated in the perikarya and dendrites of neurons, almost all neurofilaments in axons (including optic nerve axons) are phosphorylated [36,37]. Phosphorylation of NFH appears to be a major mechanism of the formation of neurofilament crossbridges, and it is deeply involved in axonal transport, axonal plasticity, and neuronal morphology [38]. A decrease in the phosphorylation of NFH was described in ON degeneration induced by experimental glaucoma in monkeys [39], and after intravitreal injection of N-methyl-D-aspartate [40], and transient retinal ischemia in mice [41]. However, to our knowledge, changes in NFHp in the diabetic ON were not previously examined. As shown herein, experimental diabetes induced a decrease in NFHp-immunoreactivity in the distal (but not proximal) portion of the ON which was prevented by EE housing. In that context, the decrease in axon number and NFHp-immunoreactivity induced by experimental diabetes and its prevention by EE housing could account for the protective effect of EE on the CTB anterograde transport. In agreement, concomitantly with a decrease in axonal transport, a significant decrease in NFHp-immunoreactivity in the porcine ON after an acute increase of intraocular pressure was described [42].

Microglia surveys the CNS environment under normal conditions, and promptly responds to neural damage through proliferative, hypertrophic, morphological, and migratory changes [43,44]. Moreover, infiltrating macrophages from the peripheral circulation enter the site of injury. Many of the same immunohistochemical markers are expressed in these two cell types which are morphologically indistinguishable from each other; therefore they are often referred to as microglia/macrophages. Microglial activation was reported in the retina at early stages of diabetes [44–46], and also in the ON from humans with diabetes [47]. The present results indicate an increased microglia/macrophages response in the distal portion of the ON from diabetic animals housed in SE, which was prevented by EE housing. Reactive gliosis is a hallmark of many neurodegenerative conditions, including diabetes [48]. Astrocytes in the CNS respond to tissue damage by becoming reactive. They migrate, undergo hypertrophy, and contribute to form a glial scar that inhibits axon regeneration. Therefore, limiting astrocytic responses represents a potential therapeutic strategy to improve functional recovery [49]. As shown herein, EE housing prevented the increase in GFAP immunoreactivity in the distal portion of the ON at early stages of experimental diabetes.

MBP is an important constituent of CNS myelin sheaths and plays a major role in myelin membrane formation and structure [50]. Therefore, MBP is a reliable index of oligodendrocyte differentiation and myelin formation. At 6 weeks of diabetes, myelin disarrangement shown by alterations and disorganization of MBP-immunoreactivity and profound myelin alterations at ultrastructural level were observed in the distal portion of the ON from diabetic animals housed in SE, whereas EE housing preserved ON myelin arrangement and ultrastructure.

While studies on structural synaptic plasticity induced by EE have focused on neuronal elements, the structural-functional plasticity of glial cells remains relatively unexplored [51]. The present results could suggest an experience-dependent plasticity of ON microglia, astrocytes and oligodendrocytes even in adult animals, and particularly in a diabetic background. In agreement, it was shown that EE housing reduces the number of microglial cells in the mouse sensori-motor cortex after photothrombotic cortical infarct [52], and that raising rats in EE decreases the effect of aging on the number and size of astrocytes, as well as on GFAP percentage in the hippocampus [53]. Moreover, it was demonstrated that EE delays the loss of myelinated fibers in the white matter of rats during normal aging [54].

It has been postulated that environmentally-induced plasticity in the brain does not simply consist of changes in different classes of cells independently; rather, interactions between neurons and glia are also altered to more optimally meet physiological and behavioral demands [55]. In this sense, although we could not ascertain whether the damage provoked by experimental diabetes, and the protection induced by EE housing primarily occurred in axons or glial elements, our results could suggest that the bidirectional communication between axons and glial cells of the adult ON can be positively affected by EE. On the other hand, the fact that axoglial alterations at the distal portion of the ON preceded RGC loss does not implies that the initial neuronal event is axonal [26], and, instead, a sublethal impairment of RGCs could render them unable to support their axons, resulting in progressive dying-back of the axon toward the cell body [56]. In this context, the prevention of retinal diabetic damage induced by EE housing [27] could also be involved in the protective effect of EE on the diabetic ON.

The mechanisms underlying the beneficial effects of EE remain unclear. Many lines of evidence support that EE increases the availability of trophic factors, which in turn mediate changes to neurons and their supporting network [11]. Although EE housing induces changes in the expression levels of a large number of genes, one group of molecules particularly sensitive to EE are neurotrophins [57, 58] which play a key role in structural and functional plasticity during development and also in the adult [59]. In particular, one of the key factors involved in the brain protection induced by EE is BDNF. Mice reared from birth in EE have higher levels of the BDNF protein in their visual cortex at P7 [60, 61]. Moreover, the acceleration of visual-cortical development in EE animals closely resembles that observed in transgenic mice overexpressing BDNF in their forebrain [62]. In SE-housed diabetic animals, a decrease in BDNF immunoreactivity was observed in the distal portion of the ON which was prevented by EE, supporting that BDNF could be one of the molecular mechanisms involved in the protective effect of EE at the ON level. In this sense, we have previously shown that EE avoided the decrease in retinal BDNF levels at early stages of experimental diabetes [27]. Therefore, although the additional involvement of other mechanisms cannot be ruled out, these results point at BDNF as a key factor in the retinal and ON protection against early diabetic damage induced by EE. In agreement, it was recently demonstrated that visual cortex BDNF participates in the recovery of vision in adult amblyopic rats induced by EE exposure [63].

At present, which components of EE (sensory, motor, cognitive, or social stimulation) are responsible for the ON protection against early diabetic damage, and to what extent is EE in animal models relevant for humans are still open questions. EE provides an increased stimulation at multiple sensory, motor, cognitive and social levels. Although most humans do experience a high degree of environmental complexity, some of the EE components can be overstimulated in humans with diseases involving neurodegeneration such as diabetes. In this sense, changes in several aspects of the lifestyle, such as regular social interactions and/or physical exercise could be recommended to humans at risk of diabetic visual damage. In this way, preservation of the visual functions through an intensified use of the sensory and motor systems, and/or social interactions could be a novel and non-invasive possibility to promote ON protection. Recently, Sale et al. [7] have raised the view of EE as an “endogenous pharmacotherapy” in which neural plasticity is not obtained by external administration of active substances, but using the environmental stimulation to enhance the spontaneous reparative potential held by the brain. Besides diabetes, a similar scenario of ON damage (i.e., microglial reactivity, astrocytosis, and loss of myelin and axons) has been described in other visual dysfunctions, such as glaucoma and ON crush. In this line, despite that more studies are required to define the mechanism responsible for the ON protection of adult diabetic animals in enriched conditions, these data support the further investigation of a non-invasive strategy such as EE as a means of promoting successful protection of the ON structure against injury, dysfunction, and degeneration.
